# Data-mining the FlyAtlas online resource to identify core functional motifs across transporting epithelia

**DOI:** 10.1186/1471-2164-14-518

**Published:** 2013-07-30

**Authors:** Venkateswara R Chintapalli, Jing Wang, Pawel Herzyk, Shireen A Davies, Julian AT Dow

**Affiliations:** 1 Institute of Molecular, Cell and Systems Biology, College of Medical, Veterinary and Life Sciences, University of Glasgow, Glasgow G12 8QQ, UK

**Keywords:** Drosophila melanogaster, Functional genomics, Ion transport, Microarrays

## Abstract

**Background:**

Comparative analysis of tissue-specific transcriptomes is a powerful technique to uncover tissue functions. Our FlyAtlas.org provides authoritative gene expression levels for multiple tissues of *Drosophila melanogaster* (1). Although the main use of such resources is single gene lookup, there is the potential for powerful meta-analysis to address questions that could not easily be framed otherwise. Here, we illustrate the power of data-mining of FlyAtlas data by comparing epithelial transcriptomes to identify a core set of highly-expressed genes, across the four major epithelial tissues (salivary glands, Malpighian tubules, midgut and hindgut) of both adults and larvae.

**Method:**

Parallel hypothesis-led and hypothesis-free approaches were adopted to identify core genes that underpin insect epithelial function. In the former, gene lists were created from transport processes identified in the literature, and their expression profiles mapped from the flyatlas.org online dataset. In the latter, gene enrichment lists were prepared for each epithelium, and genes (both transport related and unrelated) consistently enriched in transporting epithelia identified.

**Results:**

A key set of transport genes, comprising V-ATPases, cation exchangers, aquaporins, potassium and chloride channels, and carbonic anhydrase, was found to be highly enriched across the epithelial tissues, compared with the whole fly. Additionally, a further set of genes that had not been predicted to have epithelial roles, were co-expressed with the core transporters, extending our view of what makes a transporting epithelium work. Further insights were obtained by studying the genes uniquely overexpressed in each epithelium; for example, the salivary gland expresses lipases, the midgut organic solute transporters, the tubules specialize for purine metabolism and the hindgut overexpresses still unknown genes.

**Conclusion:**

Taken together, these data provide a unique insight into epithelial function in this key model insect, and a framework for comparison with other species. They also provide a methodology for function-led datamining of FlyAtlas.org and other multi-tissue expression datasets.

## Review

### Introduction

Most cells in multicellular organisms share a common genome, but improve their collective fitness by delegating specialized functions to specialized tissues. As mRNA is costly to make, genes that are particularly abundantly expressed in a tissue can provide a valuable indication of likely important functions within that tissue. Based on this premise, comparative atlases of gene expression across multiple tissues and life stages have become valuable and heavily used tools in the functional genomics arsenal [1-3]. At the simplest level, such resources allow an experimenter to establish which tissues express a gene of interest most abundantly, a necessary preliminary to a reverse-genetic work-up [4]. However, as well as allowing simple gene-by-gene lookup, such datasets allow new insights to be synthesised by data mining. For example, large microarray datasets are ideal for clustering genes by co-expression, and thence for inference of shared *cis*-acting regulatory elements [5] and gene regulatory networks [6-8]. However, there is also scope for meta-analysis of function, a relatively unexplored area. For example, it is possible to ask the question: *“which genes are uniquely expressed in the larval, rather than adult, CNS?”* This paper illustrates this methodology, using meta-analysis of tissue-specific transcriptomics datasets generated in our lab, which form the FlyAtlas.org online resource [9,10], that has quickly become one of the most widely used *Drosophila* online resources, to seek a common expression signature shared by major epithelia.

**Figure 1:**
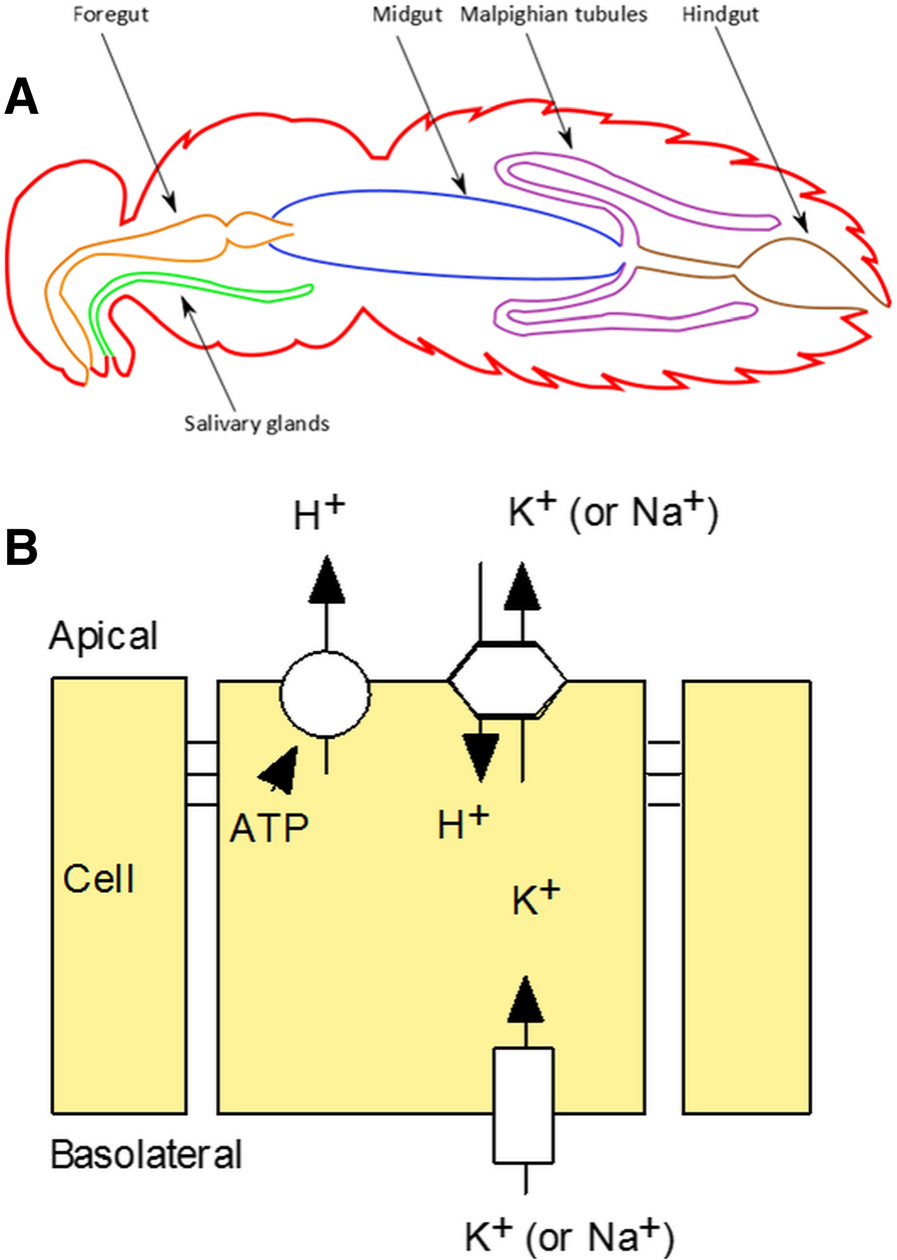
**Insect transporting epithelia and the V-ATPase hypothesis. (A)** Typical insect cross-section, after [16]. **(B)** Current dogma for insect transporting epithelia (the ‘Wieczorek model’). Transport is energized by an apical protonmotive V-ATPase, which establishes a gradient that drives an Na^+^/H^+^ or K^+^/H^+^ exchanger. These ions enter basally through unspecified mechanisms, likely to be cotransports or channels.

**Figure 2:**
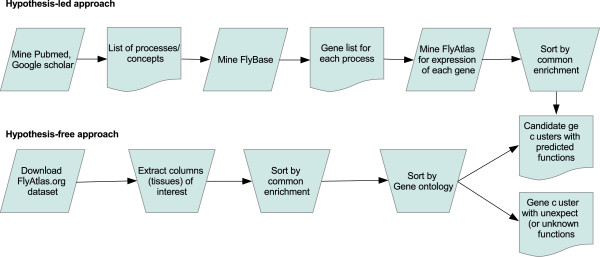
**Comparison of the hypothesis-led and hypothesis-free approaches.** The former seeks to identify genes underlying processes demonstrated experimentally, or predicted, in the literature. The latter is based on co-expression or enrichment in tissues of interest compared with other tissues, or the whole organism. Although both identify genes of interest that underly known functions, the hypothesis-free approach also identifies co-enriched genes without prior knowledge, potentially leading to unexpected research hypotheses.

The FlyAtlas.org online resource [9,10] curates Affymetrix-derived expression data (in 4 biological replicates) for each of 18 matched adult and 8 larval tissues, and one cell line, so providing unique opportunities to investigate expression across different tissues. The aim of this paper is thus to identify both the common and unique transport components across the major *Drosophila* transporting epithelia, using both a hypothesis-led approach, based on already known transport processes, and a hypothesis-free approach, based on enriched expression in one or more of these tissues. Insects make an ideal starting point for such study, because it is generally agreed that all insect epithelia are energized by an apical plasma membrane H^+^ V-ATPase (the “Wieczorek model” - Figure 1), rather than the basolateral Na^+^, K^+^ ATPase familiar to vertebrate physiologists [11,12] – although we have shown the latter ATPase also to be important [13]. Although transcriptomic abundance is not necessarily a predictor of active protein, epithelia are particularly suited to such an approach, because the relatively low turnover numbers of most transporters requires high levels of both proteins and their encoding mRNAs. We have previously shown that, across the large V-ATPase gene family, very high mRNA abundance is indeed a good indicator of functional significance in epithelia [14,15]. The concept of a core epithelial transcriptome is thus perfectly plausible, and so here we test the model by meta-analysis of larval and adult transcriptomes of the key epithelia of the alimentary canal: the salivary glands, midgut, Malpighian tubules, and hindgut (Figure 1). We adopted parallel hypothesis-led and hypothesis-free approaches (Figure 2), to maximise the unbiased discovery both of genes that underly functions already described in the physiological literature, and to uncover new co-enriched genes that might provide novel insights into epithelial function.

### Epithelial transcriptomes cluster separately from other tissues

**Figure 3:**
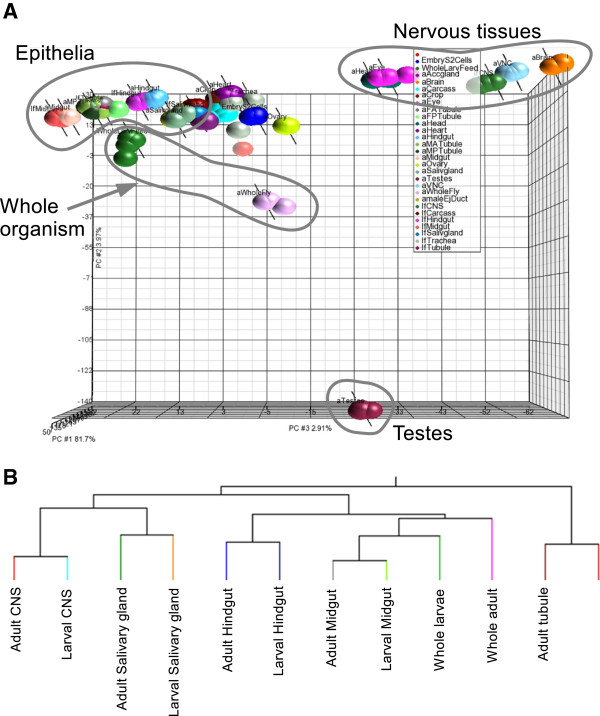
**Epithelia cluster together, and distinct from non-epithelial tissues. A**. The PCA was performed on the grouped replicates of each tissue. In a principal component (PC) all the epithelial tissues are distinctly clustered apart from all other tissues including neuronal tissues, whole fly and whole larvae. **B**. Hierarchical clustering of epithelial transcriptomes.

The first step is to establish that there is indeed a story to tell, and that epithelial transcriptomes resemble each other more than other tissues. Principal component analysis (PCA) clearly showed grouping of the epithelial tissues that was separable from neuronal or reproductive tissues, in both larvae and adults (Figure 3). This tight clustering of the 4 biological replicates of each tissue, and of the epithelial transcriptomes together and distinct from other tissues, provides broad validation for the concept that an epithelial core transcriptome is a calculable and worthwhile enterprise.

Given that epithelia sit together as a distinct group, it is logical to ask which epithelia are most closely related to each other in terms of transcriptional profile. Hierarchical clustering [17-19] confirmed that, even though most insect tissues undergo extensive remodelling during metamorphosis, the pairs of cognate adult and larval tissue transcriptomes clustered more closely together than to any other tissue. Within the hierarchy, the midgut and hindgut transcriptomes were most similar to each other, as were the tubules and salivary glands (Figure 3B). This may reflect a basic difference between absorptive (midgut and hindgut) and fluid secretory (salivary gland and tubule) epithelia, respectively. These differences are more marked than those which would have been predicted from development; the salivary glands, tubules and hindgut are ectodermal, but the midgut endodermal, in embryonic origin.

### Testing the model - is there a core epithelial signature?

The first approach adopted (Figure 2) was to profile the expression of genes underpinning functions considered to be integral to the Wieczorek model for insect epithelia, namely the V-ATPase, and putative exchangers and co-transports (Table 1). The FlyAtlas dataset allows both larval and adult tissues to be compared. Additionally, two non fluid-transporting tissues (brain and testes) were selected as out-groups, to allow comparison with the true epithelia. By inspection it is clear that, for the major classes of transporter listed in Table 1, it is possible to identify a minimal core epithelial module across most –if not all- epithelia.

**Table 1:** Distribution of key transport gene expression across epithelial and other tissues

**Gene**	**Salivary gland**	**Larval Sali. gland**	**Midgut**	**Larval midgut**	**Tubule**	**Larval tubule**	**Hindgut**	**Larval hindgut**	**Brain**	**Testis**	**Whole fly**
**V-ATPase subunits**											
**V**_**1 **_**domain**											
vha68-1 (A)	218	22	73	73	137	39	349	230	2615	159	465
vha68-2 (A)	13958	4065	4665	5247	6242	4898	5496	5099	441	353	1905
vha68-3 (A)	10	4	2	6	3	1	3	1	1	1403	109
Vha55 (B)	6275	2265	2366	3636	4016	2817	3011	3827	1856	227	1071
vha44 (C)	5309	1474	1738	1753	2131	2524	2902	3131	1023	111	619
vha36-1 (D)	6927	1937	2245	2287	3336	2417	3089	3154	1008	170	859
Vha36-2 (D)	3	2	3	2	1	1	0	0	0	716	45
Vha36-3 (D)	20	7	6	7	10	4	50	30	8	30	36
vha26 (E)	11317	3937	4330	4646	6448	3979	5075	5110	2409	777	1976
vha14-1 (F)	7440	2941	2857	3341	3210	3995	3850	3612	1657	326	989
Vha14-2 (F)	7	1	2	2	2	2	2	1	3	245	23
vha13 (G)	11129	3385	3960	3763	4963	4227	4468	4000	2318	889	2247
vhaSFD (H)	5151	2232	2332	3306	3711	3002	3324	3995	1047	589	917
**V**_**0 **_**domain**											
vha100-1 (a)	630	409	176	261	204	383	283	270	1039	156	242
vha100-2 (a)	6360	1616	1469	1655	3657	1758	3357	3184	87	83	662
Vha100-3 (a)	9	7	1	2	4	3	1	2	4	493	47
Vha100-4 (a)	15	8	725	1181	10	6	9	12	3	5	33
Vha100-5 (a)	6	2	1519	2104	419	636	423	1316	4	13	180
Vha16-1 (c)	4822	1952	3306	3163	3433	3409	3808	3350	1339	332	1308
Vha16-1 (c)	13363	5816	4646	4686	5140	4660	5211	4353	2637	656	2224
Vha16-2 (c)	3	2	1	2	3	3	1	2	2	178	12
vha16-3 (c)	6	5	2	4	3	3	3	2	2	212	19
Vha16-4 (c)	13	9	7	6	5	4	9	4	7	130	13
Vha16-5 (c)	13	8	8	2	7	5	4	4	3	318	33
vhaAC39-1 (d)	4106	1209	2203	2275	2650	1964	2671	2904	1069	198	747
VhaAC39-2 (d)	32	5	27	16	10	7	8	4	3	215	15
VhaM9.7-1 (e)	441	327	175	183	459	416	191	165	72	52	178
vhaM9.7-2 (e)	6816	2324	3437	3702	3706	3032	3451	3456	706	463	1158
vhaM9.7-3 (e)	15	9	5	14	3	2	12	6	1221	39	61
VhaM9.7-4 (e)	5	13	4	2	4	3	13	12	2	684	57
VhaPPA1-1 (c”)	8288	3139	3673	3796	5532	4187	4577	4511	1603	493	1130
VhaPPA1-2 (c”)	5	8	1	4	3	1	3	7	1	370	27
VhaAC45	8824	2883	3584	3461	4606	3690	4122	3809	1495	319	1210
**CPA exchangers**											
nhe1	1001	1338	244	245	521	442	259	226	200	136	120
Nhe2	6	10	36	16	6	51	14	85	53	12	9
Nhe3	95	61	32	60	28	86	377	185	557	15	81
Nha1	2106	57	288	374	155	38	3064	1157	8	12	99
Nha2	187	34	18	20	874	376	1653	742	10	7	45
**Selected other exchangers and cotransports**											
NKCC	2805	626	98	150	62	9	3715	886	273	116	125
Ndae1-RA/B	7	240	47	114	111	383	78	83	107	27	22
Ndae1-RC	2	14	1	3	7	6	6	1	87	60	9
Nckx30C	22	13	5	8	9	8	10	6	323	23	20
NaPi-T	2	1	2	1	2613	1490	8	5	1	0	43
Ncc60/Hsp60B	10	174	61	87	7	16	29	74	159	97	70
Prestin	75	50	373	313	339	318	266	507	55	37	92
**Na**^**+**^**,K**^**+ **^**ATPase subunits**											
Atpalpha-RC	1193	479	707	265	5427	996	3624	1255	1673	113	683
Atpalpha-RD	195	88	81	72	314	191	812	281	2489	26	213
CG3701	2	1	4	1	2	1	4	3	1	69	8
Nervana	3561	694	1327	1498	4556	2272	2922	2277	69	92	1102
nervana2	140	213	16	27	14	14	122	226	1779	114	193
nervana3	23	6	172	47	11	2	3143	7	3653	22	389
CG5250	14	2	9	11	3	2	2	5	4	256	19
**K**^**+ **^**channels**											
Ir	7480	725	506	782	1099	844	83	302	32	6	201
Irk2	235	66	117	23	805	13	4564	3856	981	19	157
Irk3	8	2	4	7	4932	2898	48	4	328	0	115
KCNQ	67	62	269	121	245	180	170	832	139	10	37
CG10465	442	494	392	354	705	610	458	478	500	410	463
CG1467	271	226	247	245	421	359	203	239			275
Shaker-RA	11	25	2	3	4	2	27	10	777	3	5
Shaker-RC	19	2	4	6	3	5	24	12	74	1	5
Shaker-RD	11	7	2	1	4	5	12	7	777	1	16
SK	1	0	1	0	0	1	0	0	0	1	0
SK-RA	28	24	46	3	6	7	9	7			6
SK-RC	41	37	11	10	7	4	68	27	700	8	34
SK (CT36054)	2	2	1	1	1	1	1	1	26	1	0
Shaw-RA	12	20	37	47	12	13	20	19	164	13	18
Shaw-RB	16	3	37	28	6	10	38	30	102	154	24
CG10830	7	181	35	19	3	5	45	26	1288	11	37
CG10440	6	2	5	6	2	16	6	5	258	4	9
Ork1	29	0	111	60	38	17	164	413	129	0	180
CG15654	2	1	3	6	2	1	27	21	0	14	21
Ih-channel (Ih)	21	7	28	29	29	16	116	35	998	47	77
**Water channels**											
Drip	7135	495	352	280	589	1024	466	937	12	312	116
CG4019	2145	100	808	493	674	1339	162	409	72	14	455
CG7777-RA	2488	247	731	310	1188	1785	570	1738	109	38	1025
CG7777-RB	15	4	8	23	6	7	5	9	1	1	116
CG17662	13	6	10	211	5	5	9	8	4	6	3
aquaporin	18	3	44	9	14	4	233	73	6	141	276
**Cl**^**- **^**channels**											
ClC-a	43	24	36	31	16	20	12	19	15	7	12
ClC-a-RD	818	88	125	261	1162	660	322	367	343	12	106
ClC-b	173	163	164	191	130	250	169	177	211	55	136
ClC-c	568	1056	292	703	1335	875	361	600	568	75	317
ClIC	1165	610	1123	587	146	153	863	803	160	74	246
CG11340	6	9	202	129	183	120	3	11	2	2	14
Best1	133	26	79	155	551	300	644	405	144	25	163
Best2	487	196	32	9	127	96	442	541	7	114	191
tweety-RB	4	2	1	2	2	1	2	4	33	1	4
tweety-RA	4	1	1	4	2	1	4	4	88	7	5
**Carbonic anhydrase**											
CAH1	13574	1389	2982	2571	978	1082	2906	3961	1566	121	478
CAH2	1061	39	625	129	516	3	118	1248	394	42	102
CG3669	2	1	1	1	1	1	0	1	1	0	0
CG3940	112	14	434	475	16	25	364	176	1329	16	193
CG18673	13	9	4	11	182	20	34	2	4	243	21
CG6074	4250	15	148	316	13	2	941	348	8	21	64
CG11284	902	636	546	942	1303	994	585	498	292	33	492
CG18672	5	0	1	5	2	1	0	0	0	3	16
CG1402	14	3	3	7	5	3	2	7	114	2	4
CG5379	8	3	1	1	14	0	1	2	23	6	5
CG10899	12	4	2	5	4	5	4	4	4	26	4
CG32968	4	28	8	9	7	2	8	14	16	1	30
CG12309	5	4	5	4	3	4	1	5	1	1145	79
CG9235	27	11	8	15	11	13	13	9	8	281	28

For brevity, the mean normalized Affymetrix signal is shown for each tissue and gene. Errors are typically 5-10% of the mean, and can be found by direct interrogation of the full dataset at flyatlas.org. Brain, testis and whole fly signals are included as non-epithelial out-groups for comparison.

In all cases, the plasma membrane isoform of the V-ATPase dominates, and a single gene is favoured for each subunit in all the epithelia studied. These genes also map precisely to those previously implicated by *in situ* hybridization and other techniques [14], increasing our confidence in the accuracy of this transcriptome-led approach. The Wieczorek model also requires an apical exchanger, possibly electrogenic [20]. Here, there is more variability; but all epithelia show very high levels of one or more of *Nhe1*, *Nha1* or *Nha2*. In the Malpighian tubule, NHA1 & 2 have previously been shown to localize to the apical plasma membrane, and to constitute the ‘Wieczorek exchanger’ [21], although Nha2 has been localized to the apical plasma membrane of the stellate (rather than principal) cell in *Aedes* tubule [22].

Basolaterally, it is important that the plasma membrane is ‘balanced’ so that the cell is not unduly stressed by the potent transport demands of the apical surface. It has been traditional in the insect literature to downplay the role of the Na^+^, K^+^ ATPase, because insect epithelia are famously insensitive to the inhibitor ouabain [23]. However, in *Drosophila* Malpighian tubule the genes encoding the α and β subunits of the Na^+^, K^+^ ATPase were very abundantly expressed [15], andthat the pump was normally protected by a co-localized OATP transporter, so conferring apparent ouabain insensitivity to a ouabain-sensitive pump [13]. Table 1 confirms that the same α and β subunit genes (*atpalpha* and *nirvana/nirvana3*) are abundantly and specifically expressed in every epithelium (Table 1), confirming the general importance of the Na^+^, K^+^ ATPase in insect epithelia.

A basolateral Na^+^ or K^+^ entry step is also required for transepithelial transport. Various cotransports have been implicated, and the Na^+^-Dependent Anion Exchanger NDAE1 [24] and Na^+^/K^+^/2Cl^-^ cotransport NKCC [25] both show enriched expression in most epithelia. However, most insect epithelia actively transport K^+^ in preference to Na^+^; and the conspicuous story in the FlyAtlas dataset is the dominance of epithelial transcriptomes by inward rectifier K^+^ channels (Table 1). Although the K^+^ channel repertoire is the most diverse in any organism, only these three channels are ever abundant in epithelia. These inward-rectifying channels would allow entry, but not exit, of K^+^, and so would provide a perfect foil to the apical exchanger. Consistent with this, secretion by the tubule is known to be inhibited by basolateral application of antidiabetic sulphonylureas such as glibenclamide, classical inhibitors of inward rectifier channels [26].

The Wieczorek model focuses on the electrogenic active transport of cations, but this is only part of epithelial function. Many transporting epithelia are specialized to move water, typically with active cation transport that energizes a passive anion flux (typically of chloride); the resulting transepithelial flux of salt drives osmotic movement of water. Although in some insect tubules chloride movement has been argued to be paracellular [27], it is reasonable to look for enriched expression of chloride and water channels across the FlyAtlas dataset. These are indeed observed across the epithelia. Although there is variability in the choice of channels, all epithelia show high levels of expression of one or more of the CLC or CLIC chloride channels; and of one of three aquaporins (there are a total of 6 in *Drosophila*).

Carbonic anhydrase is regularly found at high levels in epithelia, such as the human kidney [28]. Although the reaction it catalyses (hydration of CO_2_: CO_2_ + H_2_O < − > H^+^ + HCO_3_^-^) is reversible and spontaneous, this enzyme has a high turnover number, and is thought to be critical in providing sufficient ions for transport to occur at high rates. Although there are several carbonic anhydrase genes in the genome, only CAH1 is found at high levels in all epithelia.

Generally, then, the Wieczorek model holds for these epithelia, but the minimal core can reliably be extended to include other channels, aquaporins, exchangers and co-transports to produce a new model for insect epithelia.

### Novel gene signatures common to transporting epithelia

**Figure 4:**
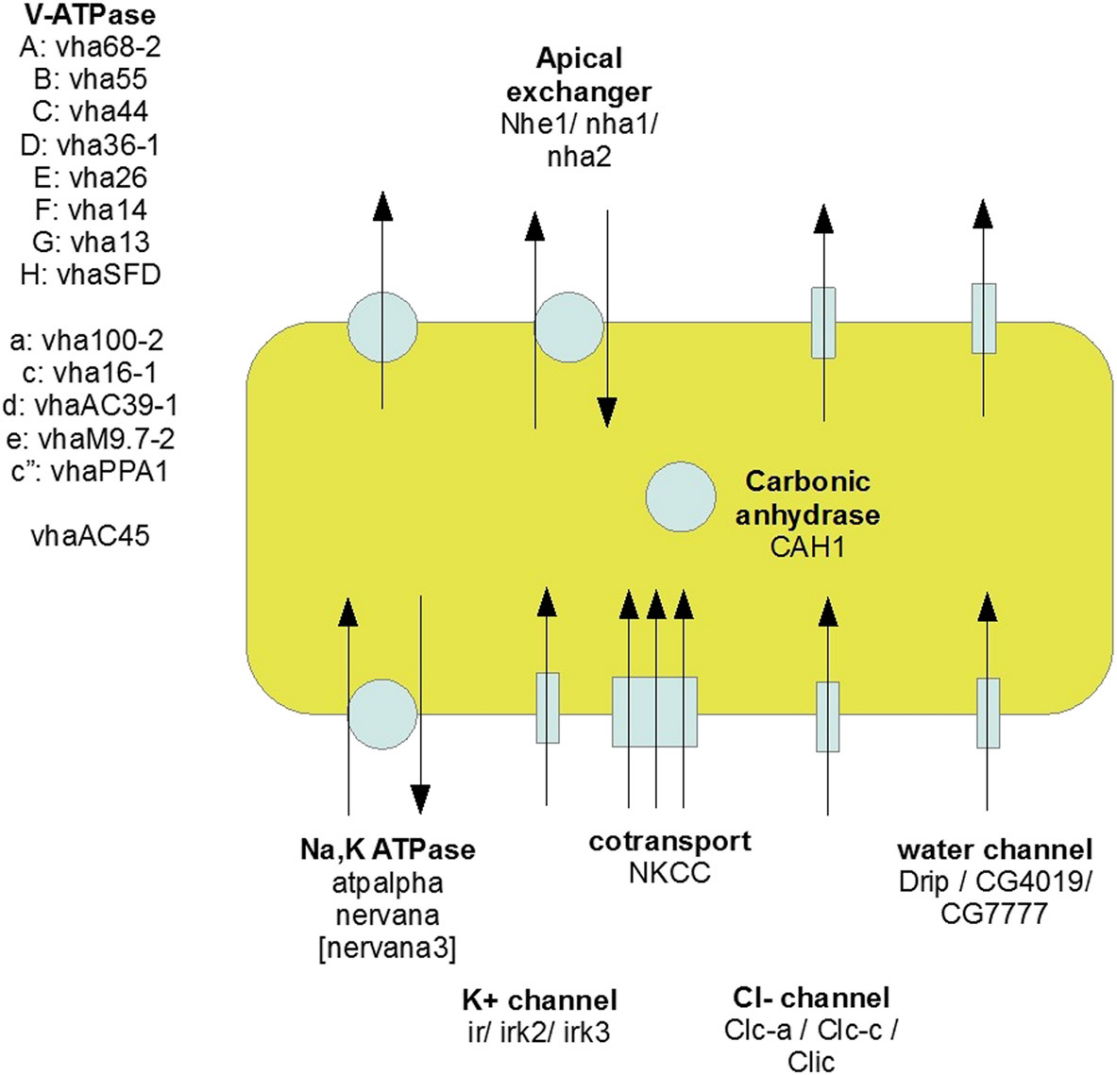
**Graphical summary of the core epithelial transcriptome from Table**1**, illustrating a common ‘core’ set of transporters shared by transporting insect epithelia.** Note that the localization (apical, basolateral etc.) is not proven by transcriptomic data, but is based on experimental physiology in previous publications.

The hypothesis-led approach confirmed the existence of a conserved epithelial core transcriptome (summarized in Figure 4). However, one advantage of global datasets is that they also permit a hypothesis-free approach (Figure 2). Are there any unsuspected commonalities between epithelial tissues, and what do they tell us about insect epithelial function? There are several potential methods to identify such genes; for example, one could identify all those genes scored by the Affymetrix software as ‘present’ in all epithelia and ‘absent’ in all other tissues. However, this would be an excessively stringent criterion, and indeed would not identify any genes in this dataset. Accordingly, we settled on a simple enrichment of RNA signal in each tissue, compared with the whole organism, and to restrict the number of hits to a manageable level, present genes with an enrichment >2.5 in all epithelial tissues (Table 2).

**Table 2:** Genes that are consistently enriched (at least 2.5-fold) across all epithelia

**Gene symbol**	**Fold change over adult whole fly**								**Description**
	**ASG**	**AMG**	**AMT**	**AHG**	**LSG**	**LMG**	**LMT**	**LHG**	
*Bowl*	**14.8**	3.2	**10.8**	7.1	**10.9**	5.6	**11.5**	5.5	transcription factor
*bru-2*	5.6	3.1	**8.8**	5.9	2.9	2.9	6.9	8.3	RNA binding, translation regulation
*Cdep*	6	4.9	4.9	3.7	4.0	4.4	5.9	4.9	Rho exchange factor activity
*CHKov1*	**18.4**	3.9	3.7	3.7	7.5	5.2	3.0	5.1	RNA-directed DNA polymerase
*Cyp12e1*	2.8	4.4	5.3	2.8	**10.9**	9.1	**12.1**	5.5	Cytochrome P450, E-class, group I
*Cyp12e1*	2.8	4.9	5.1	4.4	**12.7**	8.9	**20.3**	6.4	Cytochrome P450, E-class, group I
*Cyp9c1*	3.8	3.9	8.5	7.3	7.1	9.12	**10.1**	**16.9**	Cytochrome P450, E-class, group I
*Drip*	**63.1**	3.79	8.2	5.6	5.9	3.7	**10**	**14.6**	Aquaporin, water channel
*Hr39*	**10.3**	3.7	9.9	4.8	7.5	7.5	**13**	8.3	transcription factor, Zinc finger
*l(1)G0168*	**20.5**	2.4	3.5	3.6	**17.6**	4.0	6.2	4.8	protein targeting to Golgi
*Lola*	5.9	3.3	8.7	6.9	6.7	2.3	3.9	5.2	transcription factor, Zinc finger,
*Mitf*	5.3	3.0	2.8	4	6.7	3.1	2.8	4.7	Transcription factor, helix-loop-helix
*Msp-300*	2.9	4	2.9	4.5	5.3	7.4	4.7	5.9	Actin binding
*mthl3*	2.2	3.1	**11.3**	3.6	**12.7**	**18**	**56.7**	**41.2**	G-protein coupled receptor activity
*mthl4*	3.7	3.5	**15.6**	5.6	**24.8**	4.8	8.3	7.9	G-protein coupled receptor activity
*Nhe1*	**10.5**	2.4	5.9	3.1	**14**	2.9	4.1	2.8	N^+^/H^+^ exchanger
*Ome*	8.6	8.9	4.2	5.5	6.2	7.2	4.6	5.7	dipeptidyl-peptidase
*Pvr*	**13.1**	3.4	5.8	3.7	4.9	2.5	2.7	3.2	tyrosine kinase
*Scrib*	5.1	7.8	6.3	4.3	4.3	9.9	**15.3**	5.5	septate junction, cell polarity
*Smox*	2.3	2.4	**12.8**	3.1	2.5	4.6	4.7	3.9	protein binding, axon guidance
*Snoo*	2.8	2.8	5.3	3.1	5.4	3.1	4.9	3.9	negative regulation of dpp signaling
*Syb*	2.6	4.1	2.5	3.9	3.0	4.1	4.5	3.1	vesicle-mediated transport
*Traf-like*	2.9	**14.8**	**14.1**	4.3	3	**20.6**	**26.3**	6.7	defence response
*Troll*	**23.7**	3.7	5.9	6	3.9	7.5	5.9	4.2	EGF-like, epithelial polarity
*unc-115*	4.9	3.9	2.9	4.5	4.5	3.6	3.2	4.5	actin binding, villin; Zinc finger

Mean fold changes (relative to whole flies) across larval and adult epithelial tissues. Where more than one probe set is available for a gene, both are shown. Abbreviations: *ASG* adult salivary gland, *AMG* adult midgut, *AMT* adult midgut, *AHG* adult hindgut, *LSG* larval salivary gland, *LMG* larval midgut, *LMT* larval Malpighian tubules, *LHG* larval hindgut.

Although some of the genes mentioned identified in the consensus model (like *Drip* and *Nhe1*) also feature in this table, most do not. This is for one of two reasons. Either (as for several V-ATPase subunits), they are also expressed generally at reasonable abundance throughout the organism, so reducing the apparent enrichment; or (as for the inward rectifier channels) different epithelia select one from a restricted set, so no single gene makes the table. This approach is thus conservative in nature, but any genes that emerge are potentially of great interest. The list includes transcription factors (bowl, lola and hr39), cytoskeletal or vesicle/trafficking proteins (Msp-300, synaptobrevin), septate junctional or cell polarity proteins (such as Scrib) and a collection of cell defence genes, such as cytochrome P450s and the zinc finger protein Traf-like. The list is also enriched for some cell signalling genes, notably two enigmatic G-protein coupled receptors of the Methuselah family, and a G-protein, G_αs60A_.

It is thus interesting that the transcriptomic enumeration of the Wieczorek model for insect epithelia can be complemented by an array of further genes based on the hypothesis-free approach, and that these sit naturally in groups of epithelial determination and development, cell junctions and polarity, trafficking and defence.

### Unique gene expression patterns that delineate specialized function

Finally, having identified a common transcriptomic motif for the major transporting epithelia in insects, it is interesting to seek transcriptomic insights as to the unique roles played by each epithelium. To accomplish this, the 50 most tissue-specific genes in either larvae or adult (again, based on enrichment compared to the whole organism) for each tissue were identified (Additional file 1 Table S1, Additional file 1 Table S2, Additional file 1 Table S3 and Additional file 1 Table S4), and their functions (where known) identified from FlyBase or from the literature. Rather than a purely *in silico* exercise, generations of classical insect physiology allow the data to be interpreted in the context of the known physiology of each tissue.

### Salivary glands

As in humans, insect salivary glands are thought to produce a watery secretion containing enzymes, to aid in the maceration and initial digestion of food. Secretion is under neural control (typically by biogenic amines) [29,30]. There may also be a defence function, protecting the rest of the alimentary canal by including antimicrobial peptides in the secreted saliva. Larval and adult salivary glands do not necessarily perform identical functions, as diet can change radically in the life cycle of holometabolous insects.

Although fluid secretion is by the classical Wieczorek model (Table 1), larval and adult salivary glands use different exchangers, with Nhe1 present throughout, but Nha1 specific to the larva (Additional file 1 Table S1). For cotransports, NKCC predominates in the larva, but Ndae in the adult; and aquaporins are more prominent in the larva, suggesting an increased emphasis on fluid secretion during the active growing phase when food intake is maximal. As in the closely related blowfly (*Calliphora erythrocephala*), control of secretion in the adult is via serotonin, in which separate 5-HT receptors (5-HT2 and 5-HT7) drive secretion through two independent signaling mechanisms constituting the key second messengers cAMP and Ca^2+^ respectively [31]. However, these are not the only adult salivary gland receptors; the putative GABA/glycine receptor CG7589 is expressed at extraordinary levels. By contrast, in the larva, the only G-protein coupled receptor of any abundance is methuselah-like 4, an enigmatic receptor of unknown function.

Although saliva is traditionally considered rich in digestive enzymes, levels of amylases, proteases and peptidases were unremarkable compared with other tissues. However, lipases are strongly enriched, suggesting that saliva helps to burst cells open, rather than do assist downstream digestion. Lysozyme is virtually salivary gland specific, implying both digestive and defensive roles. Defence indeed seems to play a key role, with cecropin C also being virtually salivary gland specific. Another surprise is the relative specificity of expression of *yellow* and *yellow-d*, major components of bee royal jelly, a caste-determining secretion from the analogous hypopharangeal glands [32]. *Drosophila* lacks a royalactin gene, and does not feed its young; nonetheless, the parallel in gene expression across a broad phylogenetic range is compelling.

### Midgut

The midgut transcriptomes of larvae and adults are broadly similar (Figure 4), and are conspicuous for almost midgut-specific digestive enzymes and organic solute carriers and transporters (Additional file 1 Table S2). There is also specialization for innate immunity, as the midgut is the first highly permeable tissue encountered by incoming food (Figure 1). In this context, peritrophic membrane constituents provide a mechanical protection for the delicate midgut apical microvilli.

Perhaps most intriguing is the midgut-specific expression of vha100-4, a subunit of the V-ATPase. This is surprising because the midgut is already abundantly served by highly-expressed a-subunit genes (Table 1). In particular, the putative plasma membrane isoform, which includes vha100-2 (Figure 4), is highly expressed in midgut. The solution is that the midgut is itself a complex tissue, with multiple domains. It is thus possible that vha100-4 serves a specialized function within a geographically distinct subregion of the midgut. There are two candidate processes which involve H^+^ transport, and which do not occur anywhere else in the animal. There is a region of low pH, associated with the cuprophilic, or goblet cells in the anterior midgut [33]; and a region of high pH at the posterior midgut [34]. We speculate that this isoform is associated with one of these unique functions.

### Malpighian tubules

Although the transcriptome of the Malpighian tubule was previously described [15], it is instructive to re-examine it in the light of a much more complete microarray (Affymetrix version 2 cf. 1), and against the full FlyAtlas collections of transcriptomes, allowing unique expression to be asserted with much more authority (Additional file 1 Table S3). The tubules strictly obey the consensus transport model (Figure 4), with abundant representation of both inward rectifier potassium channels and aquaporins. Of these, *irk3* and *CG17764* are relatively tubule-specific. Otherwise, they are conspicuous for organic solute transporters, with the major families hugely represented. Of interest, many of the classical eye colour genes (e.g. *white, plum, scarlet*) are highly tubule-enriched, reflecting the role of the tubule in storing and processing pigment precursors. The tubule is also enriched for several genes associated with purine metabolism (the major route for nitrogen excretion); in particular, *rosy*[35], *urate oxidase*[36] and *5-hydroxy isourate hydrolase*. The control repertoire of the tubule has been discussed extensively elsewhere [27,37-39]; of particular note are the tissue-specific expression of the receptor for the capa neuropeptides [40,41], and one of the two cyclic GMP dependent protein kinase genes, *Pkg21D*[42,43].

### Hindgut

The ectodermally-derived hindgut is the “last chance saloon” for rescue of desirable solutes (for example, water, ions, sugars, amino acids). The hindgut also finally adjusts the osmoregulatory poise of the insect, in terrestrial insects typically by producing hyperosmotic excreta to protect against the ever-present danger of desiccation. Additional file 1 Table S4 shows that sodium regulation is conspicuous in the hindgut transcriptome, as are general substrate transporters of the OAT family. The hindgut is one of the few places in *Drosophila* where FlyAtlas reports that sodium channels of the pickpocket/ degenerin family (notably *ppk6* and *ppk12*) are detectably expressed; elsewhere in *Drosophila*, they have been implicated in mainly sensory roles [44,45]. The hindgut is known to play a key role in selective Na^+^ reabsorption – Na^+^ is a conserved ion in most herbivorous insects [46]. Ion transport peptide (ITP) acts to raise hindgut Na^+^ reabsorption through the second messenger cAMP [47-49]; consistent with this, the phosphorylation site prediction algorithms NetPhos 2.0 [50] and Disphos 1.3 [51] both predict multiple serine and at least one threonine, phosphorylation consensus in each of *ppk6* and *ppk12* (data not shown). It is also conspicuous that there are also many genes of unknown function that are selectively and strongly expressed in the hindgut, hinting at processes that are yet to be identified.

## Conclusions

Here, we review a typical data-mining workflow for expression resources such as FlyAtlas.org that allows very general, rather than single-gene, insights to be obtained, and illustrate its utility with analysis of the nature of epithelial function. Although this approach is illustrated in *Drosophila*, the results are likely to have a more general significance across the insects (and thus half of all living species), and the workflow could equally be applied to other tissues, or to mammalian systems for which authoritative expression datasets are available.

The epithelia that constitute the alimentary tract, and thus the major transport sites in the insect, have diverse embryonic origins, but still demonstrate a coherent core transport transcriptome. Remarkably, despite their diverse embryonic origins, they share a closely similar transcriptomic signature that extends beyond ion and solute transport, to epithelial specification, structure and defence.

The data presented here provide clear evidence for the generality of an extended Wieczorek model, which explains the transepithelial active transport of sodium and potassium, based on a primary electrogenic pumping of protons by a conserved plasma membrane isoform of the V-ATPase, of the Na^+^, K^+^ ATPase, previously deprecated in insect models for ion transport because of apparent insensitivity to the Na^+^, K^+^ ATPase inhibitor, ouabain [13]. These results also confuse the search for the apical ‘Wieczorek exchanger’; although we have previously shown that in tubules the recently discovered NHAs dominate [21], in other epithelia, the other major class of cation-proton exchanger, the NHEs dominate (Table 1). It may be that this diversity reflects the differing requirements of different epithelia for transporting sodium and potassium. However, with a relatively clear picture of differential expression of the CPA gene family, it should be easier to frame experimental questions to address the issue.

It is also interesting to see how individual tissues add to the basic consensus motif to achieve tasks specific to each epithelium. The salivary glands, for example, are specialized for the breakdown of cell membranes, perhaps both to aid digestion and to destroy pathogens. They are also notably controlled by 5HT by comparison with the other epithelia, and express the enigmatic yellow proteins, just like the corresponding glands in honeybees. The midgut is loaded with digestive enzymes, and (presumably) uptake transporters, and the tubules express probably the widest profile of organic solute transporters of any tissue. The hindgut emphasises sodium flux, consistent with sodium being a relatively scarce resource for phytophagous insects.

A unique combination of history and availability have made *Drosophila* the insect of choice for a wide range of investigations, and indeed the availability of a well-annotated genome sequence, transcriptomics and powerful genetic tools have more than offset its very small size. However, Drosophila melanogaster is one of perhaps 30 M insect species, and so it will be interesting to see to what extent the models developed here can be generalized. To date, the model for insect epithelia being dominated by an apical V-ATPase has not been seriously challenged, so early indications are that broad applicability is likely. Of course, the demonstration of a core transcriptomic profile for insect epithelia of diverse function and embryonic origin also begs another question: could such an approach be generalized to vertebrates?

## Competing interests

The authors declare that they have no competing interests.

## Authors’ contributions

VRC, JW and PH generated and analysed the data; VRC & JATD performed the meta-analysis and datamining; and VRC, SAD and JATD wrote the paper. All authors read and approved the final manuscript.

## Supplementary Material

Additional file 1 Contains Supplementary tables 1-4. Click here for file
