# Supplementary material for: Data-mining the FlyAtlas online resource to identify core functional motifs across transporting epithelia
Source: BMC Genomics. 2013 Jul 30;14:518. doi: 10.1186/1471-2164-14-518 (PMC3734111; doi:10.1186/1471-2164-14-518)
Supplement: Additional file 1 — Contains Supplementary tables 1-4. [file 1471-2164-14-518-S1.docx]

# Supplementary data.

# Methods

**FlyAtlas data**

The FlyAtlas resource has been described elsewhere [[9](#_ENREF_9), [10](#_ENREF_10)]. Briefly, FlyAtlas allows investigators (not necessarily skilled informaticians) to look up expression of any of 13500 genes across 27 different tissues and life stages, and so discover in which tissues a gene of interest is most abundantly expressed. Although proteins can play key roles at low abundance, the basic premise is that transcription of mRNA is energetically expensive, and so very high levels of transcription, when reported in FlyAtlas, imply a particular importance of that gene in the tissue of interest. FlyAtlas data are based on Affymetrix Drosophila genome 2.0 arrays, and with 4 independent biological replicates for each sample, producing a highly consistent dataset of broad utility.

**Informatics**

The FlyAtlas data in the public domain, either as CEL files from the NCBI Geo (accession number GSE7763), or as an Excel spreadsheet from FlyAtlas.org. The spreadsheet was downloaded, and used for subsequent analysis.

Principal component analysis was performed in Partek; other analysis was performed by sorting and filetering an Excel spreadsheet containing the entire dataset (downloadable from flyatlas.org); or by short Perl scripts. For this study, attention focussed on the major transporting epithelia of Drosophila; the salivary glands, midgut, hindgut and Malpighian tubules. Data were available both for adults and for mid-final instar larvae. Such data are amenable to both hypothesis-driven and hypothesis-free approaches. For the former, data were sorted on Gene Ontology terms that reflected the abundant physiological and biochemical literature on insect ion transport, and key articles in the area (for example: [[11](#_ENREF_11), [14-17](#_ENREF_14), [28](#_ENREF_28)]. For the hypothesis-free approach, gene lists were ordered by uniqueness of expression in particular tissues; either based on present calls in a tissue (that is, choosing genes that were called as present on 4/4 arrays for that tissue, but with as few present calls as possible across all other tissues), or on relative enrichment (average signal in that tissue divided by average signal in all tissues). The functional classes of such genes were then identified from genome annotations and gene ontology terms post-hoc. In this way, a tissue can inform the experimenter as to its unique properties, without the experimenter introducing preconceptions to the analysis.

Supplementary table 1. The top 50 genes enriched in salivary glands either specifically in adult (A) or larvae (B); and common in both (C).

| **(A) Adult Enrichment** | | **(B) Larval Enrichment** | | **(C) Common Enrichment** | | |
| --- | --- | --- | --- | --- | --- | --- |
| **Gene Symbol** | **FCA** | **Gene Symbol** | **FCA** | **Gene Symbol** | **FCA (asg/awf)** | **FCA (lfsg/wlf)** |
| CG31202 | 244 | CG32073 | 48 | sens | 160 | 65 |
| CG32984 | 185 | Muc68Ca | 47 | CG34290 | 129 | 47 |
| CG32198 | 174 | pip | 44 | Sox21b | 100 | 15 |
| 5-HT2 | 127 | pip | 42 | sage | 84 | 21 |
| CG14934 | 117 | GRHRII | 38 | CG18581 | 77 | 48 |
| CG7589 | 107 | CG12506 | 38 | nvy | 75 | 23 |
| CG12310 | 101 | CG32071 | 37 | LysP | 66 | 12 |
| CG15515 | 98 | CG9737 | 36 | eyg | 65 | 16 |
| CG6074 | 63 | y | 36 | l(3)82Fd | 65 | 11 |
| DNaseII | 55 | sesB | 36 | CG8708 | 63 | 41 |
| Drip | 50 | Eig71Ea | 36 | CecC | 57 | 50 |
| CG18088 | 49 | CG14850 | 35 | CG30371 | 53 | 17 |
| CG14880 | 45 | CG15741 | 35 | fkh | 43 | 15 |
| ine | 42 | pip | 28 | CG13461 | 36 | 34 |
| CG13946 | 41 | CG2217 | 27 | CG30411 | 36 | 12 |
| CG17121 | 39 | CG15530 | 27 | 1634865_at | 34 | 14 |
| CG5630 | 39 | CG13694 | 26 | toe | 32 | 19 |
| Buffy | 37 | CG6763 | 26 | CG15890 | 30 | 10 |
| CG31516 | 36 | CG17134 | 22 | net | 29 | 17 |
| yellow-d | 33 | CG31809 | 21 | CG13950 | 26 | 30 |
| CG5630 | 32 | PH4alphaSG1 | 21 | sage | 25 | 16 |
| Nep5 | 29 | CG31810 | 20 | CG14118 | 25 | 12 |
| CG7408 | 29 | CG4334 | 20 | GalNAc-T2 | 24 | 11 |
| GS | 27 | CG5402 | 20 | GalNAc-T2 | 24 | 15 |
| CG15094 | 26 | CG32074 | 20 | PH4alphaSG2 | 22 | 18 |
| CG8668 | 26 | CG34105 | 20 | Tie | 20 | 12 |
| CG15385 | 25 | CG34279 | 19 | p24-1 | 19 | 15 |
| 1640606_x_at | 24 | CG12715 | 18 | Gmap | 18 | 11 |
| Fsh | 24 | CG17362 | 18 | FucTA | 16 | 30 |
| CG33099 | 23 | CG33256 | 17 | CG10918 | 15 | 14 |
| CG9098 | 22 | pip | 17 | CG13947 | 15 | 30 |
| CG8483 | 22 | hoe2 | 16 | CG14252 | 14 | 13 |
| trol | 22 | CG17362 | 16 | CG30104 | 10 | 11 |
| 1633370_s_at | 22 | CG14852 | 16 | CG6225 | 10 | 14 |
| Ir | 22 | CG13445 | 16 | CG33169 | 10 | 10 |
| CG31036 | 22 | br | 15 | CG30394 | 8 | 12 |
| CG31431 | 22 | CG12508 | 15 | cry | 8 | 29 |
| CG6836 | 21 | 1637802_at | 15 | CG15743 | 8 | 15 |
| pio | 21 | Eig71Eb | 15 | Hsp70Bbb/c/a | 8 | 14 |
| CG3655 | 21 | CG13170 | 14 | CG34276 | 8 | 48 |
| NLaz | 21 | l(1)G0222 | 14 | pgant5 | 5 | 10 |
| CG6675 | 21 | CG17283 | 13 | D | 83 | 6 |
| CG4839 | 21 | CG10830 | 13 | Awh | 80 | 7 |
| wbl | 21 | pip | 12 | CG13285 | 70 | 7 |
| CG4267 | 21 | CG15404 | 12 | CG6688 | 56 | 8 |
| 5-HT7 | 20 | CG15438 | 12 | 1624816_at | 41 | 8 |
| stumps | 20 | pip | 12 | CG15822 | 38 | 8 |
| CG7510 | 19 | Cdc6 | 12 | GlcAT-P | 29 | 6 |
| CG14356 | 19 | CG31704 | 12 | CG18507 | 29 | 10 |
| 1625316_s_at | 19 | 1639017_at | 12 | CG18507 | 28 | 7 |

Supplementary table 2. The top 50 genes enriched in midgut either specifically in adult (A) or larvae (B); and common in both (C).

| **(A) Adult Enrichment** | | **(B) Larvae Enrichment** | | **(C) Common Enrichment** | | |
| --- | --- | --- | --- | --- | --- | --- |
| **Gene Symbol** | **FCA** | **Gene Symbol** | **FCA** | **Gene Symbol** | **FCA (amg vs awf)** | **FCA (lfmg vs wlf)** |
| CG15263 | 137 | CG7248 | 22 | CG10659 | 55 | 7 |
| CG31446 | 70 | 1629230_at | 16 | CG6277 | 44 | 5 |
| CG34040 | 69 | CG12951 | 11 | NPC1b | 33 | 5 |
| CG5770 | 55 | mirr | 10 | Ugt86Dh | 31 | 5 |
| CG34040 | 50 | Yp1 | 9 | Muc68E | 31 | 11 |
| CG34316 | 42 | 1638967_at | 7 | CG32054 | 29 | 9 |
| CG17930 | 42 | CG18606 | 7 | 1635270_at | 27 | 5 |
| CG6901 | 38 | CG5541 | 7 | CG33346 | 26 | 7 |
| CG42335 | 33 | CG7589 | 6 | Cyp309a1 | 24 | 5 |
| CG3739 | 30 | CG14204 | 6 | CG31720 | 23 | 5 |
| lectin-24A | 30 | grn | 5 | exex | 22 | 11 |
| CG3934 | 29 | Epac | 5 | CG13658 | 21 | 6 |
| pcl | 29 | CG4991 | 5 | CG15170 | 20 | 7 |
| CG9465 | 29 | CG9896 | 5 | Try29F | 20 | 7 |
| Vha100-4 | 28 | CG18628 | 5 | CG7381 | 19 | 5 |
| CG7025 | 27 | nimC3 | 5 | Tk | 18 | 5 |
| CG5724 | 27 | CG15816 | 5 | Myo28B1 | 18 | 7 |
| CG16997 | 27 | CG33272 | 5 | Myo28B1 | 18 | 6 |
| ninaD | 26 | CG7720 | 5 | CG8690 | 17 | 8 |
| CG17929 | 26 |  |  | CG16965 | 17 | 7 |
| CG31148 | 26 |  |  | CG31259 | 16 | 6 |
| CG16732 | 26 |  |  | CG12194 | 16 | 6 |
| CG14500 | 26 |  |  | CG17167 | 16 | 7 |
| CG8093 | 26 |  |  | Amyrel | 16 | 10 |
| CG18748 | 26 |  |  | CG18635 | 16 | 5 |
| LvpL | 26 |  |  | CG30360 | 15 | 8 |
| CG34005 | 26 |  |  | CG11318 | 15 | 7 |
| CG30272 | 25 |  |  | CG9555 | 15 | 8 |
| CG1946 | 25 |  |  | CG32053 | 15 | 8 |
| CG15533 | 25 |  |  | sens-2 | 13 | 6 |
| CG33514 | 24 |  |  | 1638487_at | 12 | 6 |
| CG34236 | 24 |  |  | CG18744 | 12 | 5 |
| CG34005 | 24 |  |  | Takl1 | 12 | 5 |
| CG2772 | 24 |  |  | lab | 11 | 7 |
| CG9981 | 24 |  |  | CG14219 | 11 | 8 |
| CG18577 | 24 |  |  | Cyp12d1-p | 10 | 9 |
| Cry | 23 |  |  | CG7912 | 10 | 6 |
| Ugt86Dc | 23 |  |  | CG12780 | 10 | 6 |
| CG32483 | 22 |  |  | CG32843 | 10 | 6 |
| CG9903 | 22 |  |  | CG4363 | 10 | 6 |
| CG8693 | 22 |  |  | Cyp6a14 | 9 | 7 |
| CG31636 | 22 |  |  | CG33337 | 9 | 22 |
| CG9826 | 22 |  |  | Cyp9f3Psi | 9 | 6 |
| CG33514 | 22 |  |  | CG42348 | 9 | 6 |
| CG12766 | 22 |  |  | LvpH | 6 | 6 |
| CG11909 | 21 |  |  | CG7408 | 5 | 6 |
| nmo | 21 |  |  |  |  |  |
| CG15423 | 21 |  |  |  |  |  |
| CG18480 | 21 |  |  |  |  |  |
| CG11149 | 21 |  |  |  |  |  |

Supplementary table 3. The top 50 genes enriched in Malpighian tubules either specifically in adult (A) or larvae (B); and common in both (C).

| **(A) Adult Enrichment** | | **(B) Larval Enrichment** | | **(C) Common Enrichment** | | |
| --- | --- | --- | --- | --- | --- | --- |
| **Gene Symbol** | **FCA** | **Gene Symbol** | **FCA** | **Gene Symbol** | **FCA(at vs awf)** | **FCA( lft vs wlf)** |
| CG32024 | 59 | CG13312 | 41 | Sr-CIV | 49 | 29 |
| CG32843 | 57 | Btd | 34 | CG33282 | 91 | 43 |
| CG13313 | 49 | Jhe | 34 | CG15408 | 68 | 27 |
| CG17636 | 46 | Jhedup | 33 | CG8837 | 85 | 25 |
| CG32023 | 42 | CG6475 | 30 | CG3285 | 63 | 22 |
| CG6602 | 42 | CG3264 | 25 | CG15406 | 32 | 20 |
| CG14694 | 39 | CG10505 | 20 | CG18095 | 85 | 54 |
| CG4484 | 34 | CG8850 | 19 | CG15279 | 35 | 22 |
| CG7144 | 34 | rdgC | 18 | Irk3 | 50 | 35 |
| CG33012 | 29 | CG14958 | 18 | CG34043 | 53 | 40 |
| CG9444 | 28 | CG13516 | 18 | Oatp58Da | 57 | 47 |
| CG10226 | 27 | CG13836 | 18 | Oatp58Db/gb | 61 | 30 |
| CG7992 | 27 | CG32626 | 16 | CG3270 | 45 | 21 |
| CG10170 | 25 | rdgC | 15 | NaPi-T | 78 | 27 |
| CG7881 | 23 | CG17646 | 14 | CG13905 | 75 | 35 |
| CG13309 | 23 | CG14949 | 13 | CG32195 | 43 | 22 |
| Hsp70Aa/b | 23 | CG14963 | 13 | st | 98 | 24 |
| CG13656 | 22 | CG3303 | 12 | CG10006 | 45 | 30 |
| CG17664 | 22 | TwdlG | 11 | CG14606 | 62 | 40 |
| CG9629 | 22 | CG7888 | 11 | CG3014 | 42 | 41 |
| CG5431 | 22 | CG15771 | 10 | CG5361 | 58 | 25 |
| Hsp70Bbb | 22 | SelR | 10 | CG6465 | 52 | 22 |
| 1638611_at | 21 | bw | 10 | Ugt86Dd | 29 | 21 |
| CG33258 | 20 | 1631349_s_at | 10 | CG14857 | 22 | 21 |
| alpha-Est6 | 20 | csul | 10 | CG17751 | 84 | 36 |
| Tsp42Eq | 20 | Or35a | 10 | CG16727 | 94 | 21 |
| p38c | 20 | cad | 10 | CG11659 | 150 | 45 |
| CG5849 | 20 | CG9062 | 9 | CG5697 | 40 | 27 |
| CG1315 | 20 | CG14856 | 9 | CG6733 | 26 | 20 |
| JhI-26 | 19 | CG6225 | 9 | CG17110 | 61 | 30 |
| CG8079 | 19 | 1638280_at | 9 | CG31106 | 48 | 25 |
| PhKgamma | 18 | ome | 8 | CG10553 | 24 | 20 |
| Hsp70Bc | 18 | CG34198 | 8 | CG31097 | 46 | 32 |
| CG7720 | 18 | Pvf1 | 8 | CG31380 | 48 | 23 |
| CG15706 | 18 | AttD | 8 | CG42235 | 62 | 33 |
| Kua | 17 | CG11897 | 8 | CG42235 | 74 | 61 |
| Cyp6a2 | 17 | CG32234///axo | 8 | CG42235 | 69 | 52 |
| CG30411 | 17 | CG4586 | 8 | CG42235 | 60 | 23 |
| CG31562 | 17 | l(3)82Fd | 8 | CG42235 | 66 | 23 |
| CG13604 | 17 | E23 | 7 | CG2187 | 33 | 28 |
| mthl14 | 17 | BG642312 | 7 | CG11889 | 26 | 33 |
| l(2)08717 | 17 | CG6364 | 7 | CG3690 | 62 | 33 |
| CG6891 | 17 | CG6293 | 7 | CG2680 | 29 | 26 |
| CG13827 | 17 | sprt | 7 | CG15221 | 39 | 40 |
| 1639729_s_at | 16 | CG30375 | 7 | CG8028 | 78 | 46 |
| Oscillin | 16 | pyd | 7 | CG14195 | 31 | 21 |
| CG42329 | 16 | CG10301 | 7 | CG18814 | 19 | 31 |
| fusl | 16 | CG7431 | 7 | 1631526_s_at | 19 | 23 |
| Fmo-1 | 16 | shn | 7 | Ugt35b | 20 | 37 |
| comm3 | 16 | CG9328 | 7 | Cyp6a8 | 31 | 20 |

Supplementary table 4. The top 50 genes enriched in hindgut either specifically in adult (A) or larvae (B); and common in both (C).

| **(A) Adult Enrichment** | | **(B) Larval Enrichment** | | **(C) Common Enrichment** | | |
| --- | --- | --- | --- | --- | --- | --- |
| **Gene Symbol** | **FCA** | **Gene Symbol** | **FCA** | **Gene Symbol** | **FCA(ahg vs awf)** | **FC(lfhg vs wlf)** |
| CG9993 | 171 | CG7906 | 63 | CG1143 | 103 | 14 |
| CG34462 | 88 | CG9021 | 54 | CG13177 | 100 | 21 |
| CG17999 | 79 | CG15615 | 33 | CG15870 | 86 | 33 |
| CG32234 | 68 | CG2157 | 31 | Cyp49a1 | 80 | 21 |
| CG34109 | 67 | CG9269 | 27 | CG4459 | 76 | 28 |
| CG34109 | 63 | aret | 26 | CG3604 | 76 | 15 |
| CG6867 | 59 | CG9702 | 25 | CG14949 | 75 | 15 |
| CG42269 | 47 | Cpr49Ae | 24 | CG32564 | 73 | 12 |
| CG4726 | 43 | CG15394 | 20 | CG7365 | 71 | 28 |
| 1639350_at | 40 | Ggamma30A | 20 | byn | 70 | 28 |
| Cpr62Ba | 39 | Esp | 19 | Cyp301a1 | 59 | 20 |
| CG3332 | 37 | CG15212 | 19 | CG13215 | 53 | 17 |
| CG13618 | 36 | CG15890 | 17 | comm3 | 52 | 19 |
| CG5639 | 34 | CG13748 | 17 | CG31530 | 51 | 55 |
| CG32234 | 32 | CG13028 | 17 | Fsh | 48 | 25 |
| yellow-b | 32 | CG14516 | 17 | Ahcy89E | 48 | 11 |
| CG9427 | 32 | Osi6 | 17 | Pkg21D | 44 | 16 |
| NepYr | 31 | aret | 17 | CG4623 | 44 | 15 |
| CG7422 | 31 | CG8303 | 15 | CG14872 | 39 | 25 |
| CG18417 | 30 | Ork1 | 15 | CG8008 | 36 | 11 |
| TwdlT | 29 | retn | 14 | CG31176 | 32 | 19 |
| CG31810 | 29 | CG13082 | 14 | ine | 31 | 13 |
| CG4660 | 28 | rpr | 14 | swi2 | 31 | 19 |
| CG11550 | 28 | SP71 | 14 | 1629538_s_at | 31 | 17 |
| CG40486 | 28 | CG15213 | 13 | CG5404 | 30 | 21 |
| CG32850 | 27 | CG14826 | 13 | CG31100 | 30 | 15 |
| CG12990 | 27 | CG9196 | 13 | CG14275 | 29 | 23 |
| CG12995 | 26 | CG15213 | 12 | Irk2 | 29 | 15 |
| CG6074 | 26 | CG9747 | 12 | CS-2 | 25 | 11 |
| CG32234 | 26 | tup | 12 | fkh | 25 | 10 |
| CG30047 | 25 | Doc2 | 12 | Rh50 | 24 | 18 |
| CG32284 | 24 | CG13313 | 11 | CG4462 | 23 | 12 |
| CG31809 | 24 | CG7802 | 11 | CG12826 | 22 | 16 |
| CG10200 | 24 | CG15201 | 11 | CG6836 | 22 | 17 |
| CG34109 | 23 | CG13228 | 10 | CG12655 | 21 | 14 |
| CG14830 | 23 | CG11147 | 10 | 1638195_at | 21 | 34 |
| CG10026 | 23 | CG5002 | 10 | CG13616 | 19 | 11 |
| CG15088 | 22 | shn | 10 | CG32397 | 18 | 15 |
| CG15822 | 21 | pip | 10 | CG16820 | 18 | 15 |
| CG3823 | 21 | Cpr49Ag | 9 | Cyp4aa1 | 17 | 20 |
| CG7888 | 21 | CG5910 | 9 | 1630614_s_at | 16 | 15 |
| CG13024 | 20 | CG32645 | 8 | CG18473 | 15 | 11 |
| CG33012 | 20 | l(2)08717 | 8 | CG31900 | 13 | 12 |
| CG32850 | 20 | CG30387 | 8 | CG33143 | 13 | 14 |
| CG17190 | 20 | tadr | 8 | CG5070 | 13 | 26 |
| CG42246 | 20 | tadr | 8 | CG10702 | 11 | 19 |
| CG5928 | 20 | CG14720 | 8 | RluA-1 | 10 | 23 |
| CG2650 | 19 | CG13217 | 8 | CG31676 | 10 | 17 |
| CG33970 | 18 | AdoR | 8 | D | 10 | 16 |
| CG14253 | 18 | CG30334 | 8 | otp | 9 | 11 |
